# Women-focused development intervention reduces delays in accessing emergency obstetric care in urban slums in Bangladesh: a cross-sectional study

**DOI:** 10.1186/1471-2393-11-11

**Published:** 2011-01-30

**Authors:** Shamsun Nahar, Morsheda Banu, Hashima E Nasreen

**Affiliations:** 1 National Institute of Preventive and Social Medicine (NIPSOM), Mohakhali, Dhaka 1212, Bangladesh; 2 Research and Evaluation Division, BRAC centre, 75 Mohakhali, Dhaka 1212, Bangladesh

## Abstract

**Background:**

Recognizing the burden of maternal mortality in urban slums, in 2007 BRAC (formally known as Bangladesh Rural Advancement Committee) has established a woman-focused development intervention, Manoshi (the Bangla abbreviation of mother, neonate and child), in urban slums of Bangladesh. The intervention emphasizes strengthening the continuum of maternal, newborn and child care through community, delivery centre (DC) and timely referral of the obstetric complications to the emergency obstetric care (EmOC) facilities. This study aimed to assess whether Manoshi DCs reduces delays in accessing EmOC.

**Methods:**

This cross-sectional study was conducted during October 2008 to January 2009 in the slums of Dhaka city among 450 obstetric complicated cases referred either from DCs of Manoshi or from their home to the EmOC facilities. Trained female interviewers interviewed at their homestead with structured questionnaire. *Pearson's *chi-square test, *t*-test and Mann-Whitney test were performed.

**Results:**

The median time for making the decision to seek care was significantly longer among women who were referred from home than referred from DCs (9.7 hours vs. 5.0 hours, p < 0.001). The median time to reach a facility and to receive treatment was found to be similar in both groups. Time taken to decide to seek care was significantly shorter in the case of life-threatening complications among those who were referred from DC than home (0.9 hours vs.2.3 hours, p = 0.002). Financial assistance from Manoshi significantly reduced the first delay in accessing EmOC services for life-threatening complications referred from DC (p = 0.006). Reasons for first delay include fear of medical intervention, inability to judge maternal condition, traditional beliefs and financial constraints. Role of gender was found to be an important issue in decision making. First delay was significantly higher among elderly women, multiparity, non life-threatening complications and who were not involved in income-generating activities.

**Conclusions:**

Manoshi program reduces the first delay for life-threatening conditions but not non-life-threatening complications even though providing financial assistance. Programme should give more emphasis on raising awareness through couple/family-based education about maternal complications and dispel fear of clinical care to accelerate seeking EmOC.

## Background

Rapid urbanization makes women highly vulnerable because of poor hygiene, over-crowding, lack of basic amenities such as water and sanitation, low availability and use of formal health services including maternity care [1,2]. In developing countries, the urban poor exhibit poorer pregnancy outcome compared to those in rural areas [3,4]. The burden of maternal mortality is especially heavy in urban slums of developing countries [5-7].

In Bangladesh, the capital alone has 3.4 million people living in the slums [4,8], where the maternal health status is very poor [4,9]. Although women living proximity of facilities with skilled medical care, 70% of them in the urban slums give birth at home with untrained traditional birth attendants [10]. These high frequencies of home delivery with insufficient preparedness, exposes poor women into higher risks and complications, along with delay in referral to facilities. Reduction in the number of maternal deaths requires timely access to effective, affordable and appropriate emergency obstetric care (EmOc) services [11].

Thaddeus and Maine [12] summarize the key concepts in understanding the causes of maternal mortality is the three delays. These are the three phases at which access to care is delayed or denied, and that lack of care leads to maternal death. The three delays are delay in seeking care, delay in reaching facility, and delay in providing appropriate care [12-16]. Delay in accessing EmOC facilities during life-threatening conditions is one of the most important factors of high maternal mortality in developing countries including Bangladesh [14-16].

An Indian study indicates that access and ability to use emergency obstetric care will have maximum impact on maternal mortality; evidence also suggests that patients who make a timely decision to seek care can still experience delay, because the accessibility of health services and receiving treatment are acute problem in the developing world [12]. Reasons include shortage of qualified staff, essential drugs and supplies, coupled with administrative delays and clinical mismanagement, become documentable contributors to maternal deaths [12,14,17,18].

Recognizing the problem, in 2007 BRAC (a non-government development organization) initiated a women-focused development intervention programme called Manoshi. Manoshi is an intensive maternal, neonatal and child health program for the urban poor of Dhaka through domiciliary and facility approach. This program provides basic maternal, neonatal and child health services through home visit conducted by a cadre of community health workers (CHW) including urban birth attendants (UBA), *Shasthya sebikas *(SS) and *Shasthya kormis *(SK) selected from the local slum area. Clean and safe delivery services are provided by UBAs and Manoshi midwives free of cost through easily accessible delivery centres (DC) established in slums. One DC serves 2,000 households with a population of 10,000 [19]. SK receives a 12-day and SS receives an 18-day basic training, followed by monthly and quarterly 1-day refresher training on antenatal, postnatal and newborn care. They provide information to pregnant women and community on danger signs of obstetric complications, when and where to seek care, treatment of minor ailments, and appropriate referral for obstetric complications. UBAs receive 6-day training on birthing care, clean delivery, basic management and referral of obstetric complications. As it is well documented that financial constraints act as an important obstacle to timely access to EmOC, Manoshi provides financial support for medicine, blood transfusion and transportation cost to poor pregnant women in slums who cannot seek EmOC timely due to lack of money. The referred women are provided with full or partial reimbursement according to their economic status. Apart from this, they also convinced the families to seek care in time, arrange transport and accompanied the women to the hospital.

Manoshi maintained the emergency referral process by developing a close partnership with government hospitals, private hospitals, and NGO hospitals. To assure the quality EmOC at fixed low prices, Manoshi has signed a memorandum of understanding with Dhaka Medical College Hospital, a government tertiary level hospital, as well as local private and NGO health facilities that have 24 hours provision of comprehensive (surgical intervention) EmOC services. The referral linkages of Manoshi are facilitated by CHWs. Once a complication is recognized at home, the women inform the Manoshi CHWs via mobile phone or personally. The CHWs reach the mothers and immediately communicate with referral Program Organizers (PO) of BRAC through cell phone to arrange transportation to the EmOC facilities with a referral slip. If a complication is recognized at DC, the SS and UBA communicate with referral PO via mobile phone, arrange transport and refer the clients to the referral facilities. Referral POs are male cadre appointed at EmOC facilities to expedite receiving treatment in the hospital. They assist in the referral facilities in admitting pregnant women, buying medicine and other logistics, arranging blood and thereby reducing the delays in receiving treatment at the hospital [20].

Despite the importance given to the referral system in preventing maternal mortality by reducing the three delays, in Bangladesh, relatively little research has addressed the issue. This study aims to explore whether the delivery centres of women-focussed development intervention programme (Manoshi) reduces delays in accessing EmOC.

## Methods

### Study design

This is a cross-sectional study conducted during October 2008 to January 2009 in catchments area of DCs in urban slums of Dhaka metropolitan areas. These slums are densely populated (about 3.4 million) and they are predominantly Muslim (98%), currently married (98%) and unemployed (75%). The housing, ventilation, water and sanitation, garbage cleaning and drainage, health and education of children are very poor. The household population are mostly young (< 30 years) and 25% are children. Educational attainment in terms of ever attending school is 56% [8]. In total, 238 DCs from 892 slums of Dhaka city were included in the study.

### Study population

The study included women who had a history of maternal complications - such as prolonged and/or obstructed labour, excessive bleeding, pregnancy-induced hypertension, pre-eclampsia, eclampsia, puerperal pyrexia, mal-position, retained placenta and referred to hospital either from home - or DCs two months before the date of data collection.

### Sample size

The required sample size was calculated with 90% statistical power and 5% of significance level using the formula for two sample comparisons of proportions. According to the Bangladesh Maternal Health Services and Maternal Mortality Survey 2001 [21] approximately 64% of women in Bangladesh faced the first delay in deciding to seek care, which required about six hours. Assuming that this proportion is reduced to 48% by the presence of Manoshi, the estimated sample included 213 complicated obstetric cases for each group. Considering the non-response rate of 5%, the total sample rounded to 450, with 225 for each group.

### Selection of sample

During October and November 2008, a total of 9,052 delivery occurred in the slums and 2199 (24.3%) were referred to hospitals for obstetric emergencies and other problems which includes twin pregnancies, previous history of caesarean section, other diseases - such as diarrhoea, vomiting, jaundice, skin diseases, vertigo. Using the referral register at DCs and the home, a list was prepared with referred cases with obstetric complications and 450 complicated women were randomly selected from the list. Types of the obstetric emergencies as mentioned by the women and family members during interview were verified with the recorded diagnosis in the discharge certificate of the hospital.

### Data collection

Fifteen trained female enumerators carried out structured interviews at the doorsteps of the respondents. Data collected in this study included information on socio-demographic characteristics, obstetric history, maternal complications, illness recognition, referral process and the three delays of seeking EmOC. The questionnaire was pre-tested in a slum not located in the catchments area of DCs. The appropriateness of language, sequencing of questions, and time required to complete the questionnaire were assessed. The enumerators and three monitors received a 5-day intensive training on the study objectives, protocols and training on sample recruitment, communication skill, and maintenance of confidentiality and privacy in research. To ensure the quality of data, a 3-layered monitoring system was developed. A field supervisor along with rotating monitors, a field manager, and a medical doctor supervised the data collection.

### Outcome measures

Three delays by types of obstetric complications:

To explore the status of three delays, obstetric complications were classified into life-threatening or non-life-threatening complications as classified by *Akhter et al. *in Bangladesh [22].

• Life-threatening complications: Haemorrhage, eclampsia and retained placenta with haemorrhage.

• Non-life-threatening complications: Prolonged and/or obstructed labour, pre-eclamptic toxaemia (PET), pregnancy-induced hypertension (PIH), and puerperal pyrexia.

### Inventory of complications and diagnostic symptoms

Researcher and field enumerators asked respondents few questions to understand the complications and its time of appearance; finally we have collected ANC card and doctors prescription to confirm the diagnosis. Table 1 described the diagnostic symptoms of the following maternal complications.

**Table 1:** Inventory of maternal complications and diagnostic symptoms

Complications	Reported symptoms and identification of complication
Prolonged labour	• True labour pain in regular interval for more than 18 hours (dawn to dusk/night)
	• Light vaginal bleeding, watery vaginal discharge during labour pain
	• Non progressing labour as cervix is not/delayed open with regular uterine contraction or
	• Foetal part is not descending due to abnormal position
	• Foetal position is okay but adequate uterine contraction is absent, need labour induction by uterotonics to deliver the baby
Obstructed labour	• True labour pain in regular interval for more than 18 hours (dawn to dusk/night)
	• Light vaginal bleeding, watery vaginal discharge during labour pain
	• Cervix is open but head arrested at certain point with adequate uterine contraction.
	• Foetal size/head/foetal part is larger than maternal pelvis, birth attendant will able see the head but would not be able to bring the baby out.
	• Uterotonic drugs fail to assist in vaginal delivery
	• Need surgical intervention (caesarean section)
Haemorrhage	• Any spout of bleeding after 28^th ^weeks of pregnancy
	• Excessive bleeding during pregnancy or at the time of labour
	• Large mass of blood clot came out from genital tract
	• Excessive bleeding like tape water after delivery with or without placental delivery
Retained placenta	• Placenta is not expelling out 30 minutes after delivery of the baby
Pregnancy induced hypertension/preeclampsia	• Blood pressure ≥140/90 mm of Hg on ANC card or doctors prescription in pregnancy
	• Sever headache
	• Neck pain
	• Blurred vision
	• Pain abdomen
	• Swelling of face/hands/legs/genital organ
Eclampsia	• Convulsion in pregnancy/delivery/after delivery
	• Fell unconscious
	• Tongue bite
	• Blood pressure ≥140/90 mm of Hg on ANC card or doctors prescription
	• Pain abdomen
	• Swelling of face/hands/legs/genital organ
Malpresentation	• Foetal part (other than head) came first during the process of normal delivery
Puerperal pyrexia	• Fever within first 10 days following delivery
	• Offensive vaginal discharge
	• Pain abdomen

[*Source of information: [23,24], and [25]]

Three delays: Time of delays was calculated as mentioned by the women and family members during interview. The minimum time required to seek care after recognition of the complication, reach the facility, and receive treatment in the facility for life threatening complications was considered. It was observed that minimum time of <1 hour was required to seek care, reach and receive treatment in the facility. Thus, time taken less than an hour was considered as no delay and ≥ 1 hour as delay. Similar method used in Bangladesh Maternal Mortality Survey 2001 [21].

• 1^st ^delay (time in decision-making) was the time interval between recognition of the complication and start for the facility to seek care. Time taken ≥1 hour to make decision to seek care was considered as delay and less than an hour considered no delay [21]. Diagnostic symptoms when proved by medical prescription, we asked three questions regarding approximate beginning time of complication, time required for recognizing and for decision making.

• 2^nd ^delay (time of arrival at the facility including time needed for transport) included the time interval from starting to reaching. Time taken ≥ 1 hour to reach facility considered as delay [21].

• 3^rd ^delay (time in receiving treatment) was the time interval between reaching the facility and the treatment received. Time taken ≥ 1 hour to receive treatment considered as delay [21].

### Statistical analysis

Comparison of background characteristics and associated factors of the delays between groups were made through chi-squares, *T*-tests, and Mann-Whitney tests (used for skewed data of the three delays).

### Ethical approval

We got approval of the Research Review Committee of BRAC Research and Evaluation Division (RED). Complete information about the study was provided verbally to the potential participants. The interviews were conducted after informed consent was obtained from the participants or their guardians (in case of unconsciousness of the participants due to eclampsia/haemorrhage). Strict confidentiality was maintained about the identity of the respondents.

## Results

### Sample characteristics

Of the 450 women interviewed, three-quarters were aged 20-35 years. Women referred from home were two years older than the group referred from DCs. The respondents were predominantly Muslim and married. Almost a third of the women had never been to school. Both groups had 4 years of schooling on average. Around 12% of the women were involved in income generation, mostly as domestic workers. Around 50% of the women were primiparity. The mean number of children born in both groups was two. Grand multi-party and mean birth interval were found to be higher among women referred from home. Approximately one-fourth of women had experienced child death and 13% had a history of stillbirth. The proportion of stillbirth and infant death was almost similar in both the groups (Table 2). Approximately 57% of the women received four antenatal care (ANC) visits. The mean number of ANC visits was five in both groups and the majority received ANC from BRAC health providers (table not shown).

**Table 2:** Socio-demographic and obstetric characteristics of study population (%)

Characteristics	Referred from DC N = 225	Referred from home N = 225	Total N = 450	p value
Age (years) (%)				
Below 20	30.2	13.3	21.8	
20 - 35	65.8	82.7	74.2	
35 or more	4.0	4.0	4.0	
Mean age (±SD)	23.0 (5.5)	25.2 (5.8)		<0.001
Religion (%)				
Islam	98.2	98.2	98.2	
Others	1.8	1.7	1.8	
Marital status (%)				
Married	99.1	97.3	98.2	
Separated	0.9	2.7	1.8	
Educational status (%)				
No schooling	27.2	28.4	27.8	
Primary	39.6	39.4	39.5	
Secondary	28.4	24.4	26.5	
Secondary and above	4.8	7.8	6.3	
Mean year of schooling (±SD)	4.2 (3.3)	4.1 (3.6)		0.795
Involved in income generation (%)	8.4	16.4	12.4	0.015
Parity (%)				
None	0.0	0.4	1.2	
1	53.8	44.0	48.9	
2-3	30.2	34.7	32.4	
4-5	9.3	15.6	12.4	
6 or more	6.7	5.3	6.0	
Mean number of children ever born (±SD)	2.1 (1.7)	2.3 (1.7)		0.231
Birth spacing (interval between index and last child (Yrs) (%)				
Less than 2 years	9.3	4.9	7.1	
3-5	17.8	16.4	17.1	
5 years or more	72.9	78.7	75.8	
Mean years of birth interval (± SD) (Excluding primigravida)	4.6 (2.7)	6.4 (3.6)		< 0.001
History of child death (%)				
No death	73.8	75.8	74.2	
Stillbirth	12.0	13.0	12.7	
Infant death	11.0	10.0	10.6	
≥ 2 years	3.2	1.2	2.5	

### Obstetric complications of the index pregnancy

Of all the obstetric complicated cases, 82% respondents experienced complications during delivery, 11% during antepartum, and 7% in postpartum periods. Eighty-five percent of women had faced single complication, 15% women had multiple complications ranged 2-3; and the mean number of complications per women was 1.2. The obstetric complications were grouped into life-threatening and high-risk complications. Almost a quarter of women had life-threatening complications. Of the life-threatening complications, haemorrhage constituted the highest rank while prolonged and/or obstructed labour was the most frequently observed non-life-threatening complication. Comparing the two groups, more women from the community were referred for life-threatening complications (Table 3) and the family members did not seek care until they felt the condition was a life-threatening (figure not shown).

**Table 3:** Distribution of referred women by types of complications (%)

Types of complications	Women referred from		
	
	Delivery Centre N = 225	Home N = 225	Total
Single life-threatening complications			
Haemorrhage	6.2	16.9	11.6
Eclampsia	2.2	6.1	4.1
Multiple life-threatening complications			
Haemorrhage with PIH/PET/breech presentation	1.8	3.6	2.7
Eclampsia, haemorrhage/PIH	-	2.2	1.1
Retained placenta with haemorrhage	4.9	2.7	3.8
Retained placenta with PET	1.8	-	0.9
Single non-life-threatening complications			
Prolonged and/or obstructed labour	60.4	32.0	46.2
Pre-eclampsia	1.3	6.7	4.0
Pregnancy induced hypertension	7.2	18.2	12.7
Multiple non-life-threatening complications			
Prolong labour with PIH/PET/breech presentation	13.8	10.7	12.2
Puerperal pyrexia	0.4	0.9	0.7

*PET = Pre-eclamptic toxaemia; PIH = pregnancy induced hypertension

### Referrals: Places, decision makers, transportation and escorts

As mentioned before, the Manoshi CHWs referred women to assigned EmOC facilities. Most of the women went to the assigned facilities, except for a very few referred cases from home went to other facilities (not assigned). In both groups, husbands made all decisions in half of the cases (48% and 51%). One-fifth of the women from the DCs and one in every 10 women referred from home decided themselves to seek care during emergency. The majority (81%) of the women referred from DCs were accompanied by the CHWs, while 39% of the referral cases from home were accompanied by the CHWs. Almost all (98%) of the respondents used auto-rickshaw or rickshaw to reach the facility.

### Three delays and time taken for events

First delay (≥1 hour) was found to be higher among women referred from home (73.3%) than the women referred from DCs (66.2%). The overall median time required to make a decision to seek care was 9.7 hours among the women who were referred from home while it was 5.0 hours for women who were referred from the DCs. However, the median times to reach a facility and to receive treatment were found to be similar in both groups (Table 4). On average, around 62% reached hospital within an hour and 83% received treatment before an hour (Table not shown). Median time for making decision for referral varied by types of complication. First delay was not occurred in the case of life-threatening complications referred from DC and least time taken in case of eclampsia (0.3 hour) followed by retained placenta (0.6 hour) and haemorrhage (0.9 hour), while it was 2.2 hours to 2.9 hours among women referred from home. The median time for making decision among women referred from DC for life-threatening and non-life-threatening conditions was significantly shorter than that of those who referred from home (Table 5). Since there was no significant difference between the second and third delay between the groups, no analysis was done with these delays and types of complications.

**Table 4:** Time taken for events of "Three delays"

Three levels of delay	Median time (hrs) taken for events		p value
		
	Delivery centre	Home	
Making decision to seek care (n = 313)	5.0	9.7	< 0.001
Reaching the facility (n = 256)*	1.6	1.8	0.051
Receiving treatment (n = 61)*	1.3	1.2	0.371

* Median time among those who made delay

**Table 5:** Time required for decision making to seek EmOC care by complications

Complications	Median time (hours) for decision-making process to seek care		
	
	Delivery Centre N = 225	Home N = 225	P value
Life-threatening complications			
• Eclampsia	0.3	2.9	
• Haemorrhage	0.9	2.3	
• Retained placenta with haemorrhage	0.6	2.2	
• Haemorrhage with PIH/PET/breech presentation	0.6	3.5	
Median time (hours)	0.9	2.3	0.002
Non-life-threatening complications			
• Pre-eclampsia	4.2	6.6	
• Prolonged and/or obstructed labour	3.5	11.3	
• Prolong labour with PIH/PET/breech presentation	2.8	5.2	
• Pregnancy induced hypertension (PIH)	1.0	2.0	
• Puerperal pyrexia	0.4	7.0	
Median time (hours)	2.8	7.5	< 0.001

*PET = Pre-eclamptic toxaemia; PIH = pregnancy induced hypertension

### Reasons for delay in making decision (first delay)

**Figure 1:**
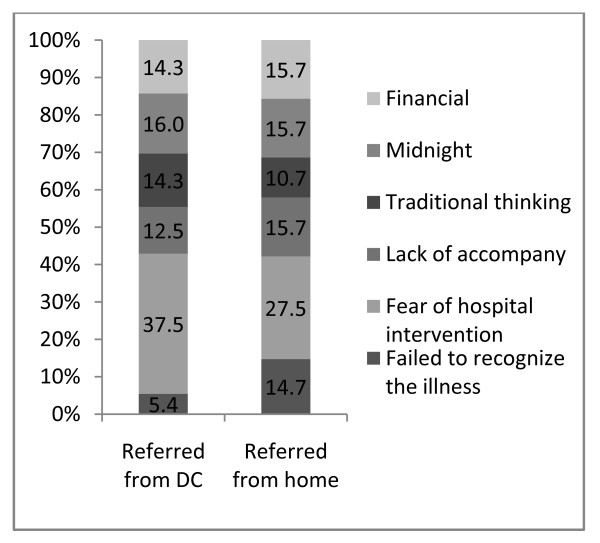
Reasons for delay in making decision for seeking care by places of referral (%)

Figure 1 presented that in both the groups, an average of one-third mentioned fear for medical intervention as the main cause. Another important reason was the inability to judge the graveness of complications, which was found among 14.5% of cases who were referred from home and 5.4% of cases referred from DCs. The frequency for other mentioned reasons for delay in making decision to seek care was found to be similar in both groups, such as lack of money, complications arose at midnight, and traditional belief or conservativeness.

### Financial assistance of Manoshi and first delay

The average cost of delivery ranged from BDT 1001 to 5000 (≈US$ 14.5-72.3, conversion rate US$ = 69.1 BDT, July 2009). The mean expenditures shared by Manoshi in both groups were around BDT 2500 (≈US$ 36.1). The findings depicted that approximately 88% (n = 397) women received financial support from Manoshi. Among them, irrespective of the type of complications (life-threatening and non-life-threatening) the women who were referred from DCs took significantly less time to decide to seek care than the women who were referred from home (0.9 hours *vs*. 2.2 hours, p = 0.006 and 3.1 hours *vs *6.1 hours, p = 0.008) (Table 6).

**Table 6:** Median time for first delay with financial assistance by status of complications

Status of Complications	Median time for first delay in hours		
	
	Received financial assistant		
	
	Delivery centre	Home	P value
Life-threatening complications (n = 112)	0.9 (n = 38)	2.2 (n = 74)	0.006
High risk complications (n = 275)	3.1 (n = 169)	6.1 (n = 106)	0.008

### Factors influencing the first delay

The delay in decision making to seek care was higher among older mothers, multiparity and mothers who were not involved in income-generating activities. The community understanding of severity of maternal condition significantly influenced the first delay (p < 0.001). Other background variables such as education, ANC visits, and history of death of previous children and gender role in decision-making did not have any significant influence on the first delay (Table 7).

**Table 7:** Factors influencing the first delay

		**1**^**st **^**delay**		
		
	Factors	DC (%)	Home (%)	p
Age	≤ 25 yrs	75.8	59.8	0.003
	> 25 yrs	24.2	40.2	
Education	No education	25.5	28.7	0.611
	Literate	74.5	71.3	
Involved in income generation	Yes	0.6	18.3	0.001
	No	94.0	81.7	
Husbands education	No education	27.5	25.6	0.798
	Literate	72.5	74.4	
Past obstetrical problem	No history of death child	76.0	79.1	0.215
	History of child death	24.0	20.9	
ANC visits	<4 ANC visits	28.2	31.1	0.621
	≥4 ANC visits	71.8	68.9	
Parity	Primiparity	59.1	45.1	0.017
	Multiparity	40.9	54.9	
Obstetric complication	Life-threatening	13.4	35.4	<0.001
	Non-life-threatening	86.6	64.6	
Decision maker	Self	18.8	19.5	0.887
	Husband and relatives	81.2	80.5	

	N	149	164	

*p = p value; DC = Delivery centre; ANC = Antenatal care

## Discussion

The study reveals that the first delay was longer compared to the second and third delays that eventually show delay in decision to seek care, which is one of the most important factors contributing delays in accessing EmOC. The first delay was longer among those who were referred from home compared to those who were referred from the DCs. The median delay between recognizing complications and deciding to seek care was significantly higher in the case of home referral than DC referral (9.7 hours vs 5.0 hours). This is consistent with the findings of the previous studies in Bangladesh and India documenting that women took 6 hours and 8 hours in making decision to seeking care during the obstetric complication [21,26]. In our study, the time to reach a facility was 1.6 hours and 1.8 hours for DC referral and home referral respectively; which is quite similar to a study in Matlab, Bangladesh where the median time to reach facility was 1.3 hours [13]. In the current study, in case of life-threatening conditions, the time required to make a decision was 0.3-0.9 hours while in case of non-life-threatening complications, the median delay was several times higher (1.0-4.2 hours). The median time for making a decision among women referred from DCs was significantly shorter than that of those who were referred from home for both life-threatening and non-life-threatening conditions. This indicates that the ability to judge the graveness of the complications of pregnancy by the Manoshi UBA and other CHWs helps reduce the time to make decision to seek EmOC. Similar situation has also been observed elsewhere [13,21]. The present study reveals that of the women who sought treatment for complications, around 64% reached the facility within an hour and almost 80% received treatment before an hour. These results suggest that accessing facilities and getting treatment are not a problem in urban areas in Bangladesh. Instead, the problems are concentrated around the delay in making the decision to seek care as supported by the findings of Bangladesh Maternal Health Services and Maternal Mortality Survey in 2001 [21].

The study elucidated several reasons for the delay in making the decision to transfer women, the main being the fear of medical interventions and the lack of money. Studies from different developing countries reported similar findings as the current study [13,14,27]. The discussions essentially indicate that most delays are multi-causal and that the community people are not aware of the danger signs of pregnancy, thus not being prepared for an emergency.

The decision-maker is considered to have influence on the aforementioned delays. In most cases, the male partner played the key role in decision-making [15,28,29]. Studies from different African countries also reported similar findings [30,31].

The socioeconomic and obstetric indicators have influence on the first delay between the two groups; it was observed that the delay was significantly higher in home referral among adult multiparity women. This may be because adult women with previous delivery experience made them reluctant to seek care from facilities. A study in Nigeria showed that parity has an influence on delay in the use of maternity care [32]. In inclusion, results of various studies in Bangladesh [33], Pakistan [34], Nepal [35], and Nigeria [31] showed that education and ANC visits had influence on the first delay. However, in our study, this association was not justified, most likely because the women of the comparison groups had similar characteristics and came from similar socio-environmental background. Delay in making decision to seek care was significantly higher among women who were referred for non-life-threatening complications. It is possibly due to the community understanding regarding maternal complications. This finding is also similar to that of another study conducted in Matlab, Bangladesh [13].

Although the cost of government services for maternity care is subsidized, they eventually escalate because of the expenditure for direct treatment cost (such as medicines, laboratory investigations, and blood transfusion), indirect costs (combined expenses of travel and food), and the high cost of maternity care. All these act as a barrier to timely seeking care [27,36,37]. Our study consistently indicates that the lack of money is a reason for the delay in deciding to seek care and first delay was significantly higher among women who were not involved in income generation. Considering financial assistance provided by Manoshi, irrespective of type of complications, the time taken for decision-making was significantly reduced. Consistent with studies in India [16] and Nepal [38], this finding elicits that providing financial incentive had an influence on receiving EmOC services.

These findings have important implications. If maternal and neonatal health is to be improved, the three delays must be addressed, and services should be more easily accessible to the mothers. Obviously, poor mothers cannot access these services easily. Therefore, the community must recognize the importance of this service, realizing that it not only benefits the mothers but the community as a whole.

As this was a cross-sectional study some limitations were unavoidable. Time calculations for three delays were collected through recall of the women and their close attendants. As a result, a recall bias could not be avoided. Furthermore, the selection bias in the groups studied was inevitable because of the lack of slums in Dhaka where the Manoshi service is not provided. Although Manoshi provided homestead service for ANC, delivery care was only providing by DC by CHW. Recognizing the severity of illness might be different depending on the place of delivery as home deliveries were attended by traditional birth attendants or by relatives.

## Conclusions

Delivery centre of Manoshi reduces the first delay for life threatening conditions. However, delay in decision-making to seek care still remains long in the cases of non-life-threatening complications. Even though, the programme provided financial support for medicine, blood, and transport. First delay was significantly higher among elderly women, non-life-threatening complications, multiparity and who were not involved in income generation. The reasons for delayed decision include community's fear of medical intervention, inability to judge maternal condition, traditional beliefs, and financial constraints. The husband played colossal role in making the decision to seek care. Therefore, along with the Manoshi delivery centre's assistance, the programme should give more emphasis on raising awareness through couple/family-based education regarding maternal complications and dispel fear of clinical care to accelerate seeking EmOC.

## List of abbreviations

ANC: Antenatal care; BDT: Bangladesh taka; BRAC: Formally known as (Bangladesh Rural Advancement committee); CHW: Community Health Worker; DC: Delivery Centre; EmOC: Emergency Obstetric Care; MNCH: Maternal, Neonatal and Child Health; NGO: Non-governmental Organization; UBA: Urban Birth Attendant; PO: Program Organizer; PIH: Pregnancy-induced Hypertension; PET: Pre-eclamptic Toxaemia; SS: S*hasthya sebika; *SK: S*hasthya kormi*.

## Competing interests

The authors declare that they have no competing interests.

## Authors' contributions

All authors (SN, MB, HEN) participated in planning, conceptualizing of the research questions, and designing the study. MB and HEN conceptualized and designed the study. MB was responsible for organizing the field activities and retrieving the data. SN and MB analyzed the data and drafted the manuscript. All authors participated in interpreting data and critically revising the manuscript for important intellectual content. SN and HEN made critical revision of the manuscript. All authors read and approved the revised manuscript.

## Pre-publication history

The pre-publication history for this paper can be accessed here:

http://www.biomedcentral.com/1471-2393/11/11/prepub
